# Coronary artery bypass surgery in patients with low ejection fraction: short and mid term outcomes according to the associated risk factors

**DOI:** 10.1186/1749-8090-8-S1-P99

**Published:** 2013-09-11

**Authors:** M Akyuz, B Lafci, U Yetkin, M Bademci, B Ozpak, I Akyildiz, B Ozcem, A Gurbuz

**Affiliations:** 1 Izmir Katip Celebi University Ataturk Training and Research Hospital, Department of Cardiovascular Surgery, Izmir, Turkey; 2 Tekirdag State Hospital Dept. of Cardiovascular Surgery, Tekirdag, Turkey; 3 Izmir Katip Celebi University Ataturk Training and Research Hospital, Department of Cardiology, Izmir, Turkey

## Background

Left ventricular dysfunction increases the rate of mortality and morbidity after coronary artery bypass grafting (CABG).

## Methods

Thirty seven consecutive patients with low ejection fraction (EF) who underwent CABG in our department were analysed retrospectively (8 women and 29 men, mean age 62,32 ± 10,86 years, range 40 to 78 years). Eight patients underwent off-pump bypass.

## Results

Twenty-two patients were diabetic (%59.5), 24 patients had hypertension (64.9%), 14 patients had hyperlipidemia (%37.8), 27 patients had a history of smoking (%73), two patients had chronic obstructive pulmonary disease (%5.4), six patients had peripheral artery disease (%16.2), one patient had a history of cerebrovascular accident (%2.7), one patient had a history of intracardiac defibrillator implantation (%2.7), three patients had percutaneous coronary intervention (%8.1).

## Conclusion

Risk factors should be evaluated for the short and mid-term outcomes of patients with low EF undergoing CABG.

